# *De novo* transcriptome based insights into secondary metabolite biosynthesis in *Malaxis acuminata* (*Jeevak*)–A therapeutically important orchid

**DOI:** 10.3389/fpls.2022.954467

**Published:** 2022-10-18

**Authors:** Paromik Bhattacharyya, Tanvi Sharma, Abhinandan Yadav, Lucy Lalthafamkimi, Ritu, Mohit Kumar Swarnkar, Robin Joshi, Ravi Shankar, Sanjay Kumar

**Affiliations:** ^1^Biotechnology Division, Council of Scientific and Industrial Research-Institute of Himalayan Bioresource Technology, Palampur, Himachal Pradesh, India; ^2^Studio of Computational Biology & Bioinformatics, The Himalayan Centre for High-throughput Computational Biology (HiCHiCoB, A BIC supported by DBT, India), Council for Scientific and Industrial Research (CSIR)-Institute of Himalayan Bioresource Technology (CSIR-IHBT), Palampur, Himachal Pradesh, India; ^3^Agrotechnology and Rural Development Division (ARDD), CSIR-North East Institute of Science and Technology, Jorhat, Assam, India; ^4^Academy of Scientific and Innovative Research (AcSIR), Ghaziabad, Uttar Pradesh, India; ^5^Chemical Technology Division, Council of Scientific and Industrial Research-Institute of Himalayan Bioresource Technology, Palampur, Himachal Pradesh, India

**Keywords:** *Ashtvarga*, *Ayurveda*, eugenol, *meta*-Topolin (*m*T), transcriptome, orchid, β-sitosterol

## Abstract

*Malaxis acuminata* D. Don [=*Crepidium acuminatum* (D. Don) Szlach.] is an endangered medicinal orchid of the *Ashtvarga* group of plants in *Ayurveda* (Indian system of traditional medicine). Using a combination of aromatic cytokinin [*meta*-Topolin (*m*T)], plant biostimulant (chitosan), auxin [indole-3-butyric acid (IBA)], and a phenolic elicitor [phloroglucinol (PG)], plants of *M. acuminata* were regenerated *in vitro* for mass multiplication. The present research reveals the first-ever transcriptome of *M. acuminata*. A total of 43,111 transcripts encoding 23,951 unigenes were assembled *de novo* from a total of 815.02 million reads obtained from leaf and pseudobulb of *in vitro* raised *M. acuminata.* Expression analysis of genes associated with β-sitosterol and eugenol biosynthesis in leaf and pseudobulb provided vital clues for differential accumulation of metabolites in *M. acuminata*. Ultra-performance liquid chromatography (UPLC) confirmed higher amounts of β-sitosterol and eugenol content in the leaf as compared to the pseudobulb. Differential expression of transcripts related to starch and sucrose metabolism, plant hormone signal transduction, diterpenoid biosynthesis, phenylalanine metabolism, stilbenoid, diarylheptanoid, and gingerol biosynthesis suggested the operation of differential metabolic pathways in leaf and pseudobulb. The present research provides valuable information on the biosynthesis of secondary metabolites in *M. acuminata*, which could be used for advanced metabolite bioprospection using cell suspension culture and bioreactor-based approaches. Data also suggested that leaf tissues rather than pseudobulb can be used as an alternate source of bioactive metabolites thereby shifting the need for harvesting the pseudobulb. This will further facilitate the conservation and sustainable utilization of this highly valued medicinal orchid.

## Introduction

In the recent times, phytomedicines have attracted increased attention for their use in the treatment of chronic ailments (Golechha, 2020). Medicinal plants display distinct bioactivities which enable their use in the food, cosmetic, and pharmaceutical industries (Dogan et al., 2014; Mollova et al., 2020; Kibar, 2021). Amongst various medicinal plants used in herbal preparations, orchids deserve special mention (Hossain, 2011; Gantait et al., 2021). Interestingly, orchids occupy a prominent position in the traditional Indian system of medicine “*Ayurveda*”. Categorized amongst the “*Ashtvarga*” group of eight medicinal herbs, *Malaxis acuminata* D. Don [=*Crepidium acuminatum* (D. Don) Szlach.] or “*Jeevak*”- a prized medicinal orchid figures out prominently (Kaur and Bhutani, 2010). The dried pseudobulb of *M. acuminata* contains rich reserves of various bioactive compounds which have antioxidant, cardioprotective, and anti-aging properties (Bose et al., 2017a; Giri et al., 2017; Balkrishna et al., 2018). Also, the pseudobulb of *M. acuminata* forms an important ingredient of “*Chyawanprash*”- an age-old health tonic marketed by various pharmaceutical brands across the globe (Bose et al., 2017a; Balkrishna et al., 2018). Recent reports on *M. acuminata* reveal an alarming scenario with high rates of anthropogenic intrusions and the destruction of natural habitats (Cheruvathur et al., 2010; Bose et al., 2017b). With a rapid change in the global climatic patterns in the past few decades, significant changes have been recorded in the habitat and distribution of plants occurring in various biodiversity hotspots globally amongst which the Himalayas deserve special mention. Based on the habitat and species distribution data, Conservation Assessment and Management Plan-World Wide Fund for Nature has categorized *M. acuminata* as “vulnerable” whereas the Convention on International Trade in Endangered Species of Wild Fauna and Flora has included *M. acuminata* within the Appendix II of endangered species (Lohani et al., 2013).

However, in spite of its high medicinal potential, there exists a persistent information gap with respect to the secondary metabolite profile of *M. acuminata* and the mechanisms regulating their biosynthesis. With habitat distribution in inaccessible regions of Himalayan, sub-Himalayan, and trans-Himalayan mountain ranges, collection of *M. acuminata* is often challenging and difficult (Bose et al., 2017a; Giri et al., 2017). In recent times, comprehensive advancements have been made in the research domain of plant tissue culture (PTC) with special reference to medicinal plants and orchids (Gantait et al., 2021). PTC is being recognized as an essential biotechnological tool not only for the supply of quality planting material (QPM) necessary for therapeutic bioprospection of metabolites but also for the sustainable replenishment of endangered germplasm of plants with high medicinal importance (Nalawade et al., 2003; Espinosa-Leal et al., 2018; Niazian, 2019). However, in order to modulate and selectively enhance metabolite production within *in vitro* systems, prior knowledge about the biosynthetic pathways, metabolic fluxes and regulatory mechanism involved in the metabolite biosynthesis is a principle prerequisite that can be obtained by the next generation sequencing (NGS) based transcriptomic approach (Han et al., 2016; Yamazaki et al., 2018; Shih and Morgan, 2020).

The emergence of NGS in the recent past has offered a feasible alternative to researchers for analyzing the genomes and transcriptome data of non-model plant species including orchids (Dhiman et al., 2019; Sharma et al., 2021). Transcriptomes of medicinal orchids, such as *Fontainea picrosperma* (Shan et al., 2021), *Dendrobium officinale* (He et al., 2020; Zhang et al., 2021), *Dactylorhiza hatagirea* (Dhiman et al., 2019), and *Gastrodia elata* (Shan et al., 2021) have been sequenced and assembled. Transcriptome profiling has provided vital molecular insights into the genes involved in metabolite biosynthesis in prized non-model medicinal plants (Yamazaki et al., 2018; Shih and Morgan, 2020). Furthermore, NGS approaches have facilitated the generation of large simple sequence repeat-expressed sequence tag (SSR-EST) datasets, especially of the non-model medicinal plants with negligible or none genomic data available (Morozova et al., 2009; Simon et al., 2009; Gai et al., 2012).

In the past few decades, a few coordinated research endeavors have contributed critical insights into the pharmacological and phytochemical constitution of *M. acuminata* (Suyal et al., 2020). However, to date, no information exists to elucidate the metabolic pathways involved in the biosynthesis of the key secondary metabolites of medicinal importance. Before the advent of NGS platforms, the identification and generation of SSR and ESTs were widely used for the understanding of biosynthetic pathways in non-model plants including orchids (Li et al., 2010; Sun et al., 2010). However, to the best of our knowledge, no information exist on the SSR and EST data at the National Centre for Biotechnology Information (NCBI) Gene Bank database.

With this background, the present study was carried out to analyze the comparative transcriptome profiles of the leaf and pseudobulb tissues of *M. acuminata* and their correlation with target metabolites, β-sitosterol, and eugenol, content. Data thus generated can further be utilized for metabolite up scaling by application of relevant biotechnological tools.

## Materials and methods

### Plant tissue culture of *M. acuminata*

Plants of *M. acuminata* were sampled from the natural habitat of upper Shillong (Meghalaya), India, and were maintained in the greenhouse of Council of Scientific and Industrial Research (CSIR)- Institute of Himalayan Bio-resource Technology (IHBT), Palampur, Himachal Pradesh. Transverse thin cell layer (*t*-TCL) explants were used for micropropagation of *M. acuminata* in Murashige and Skoog medium (Murashige and Skoog, 1962) supplemented with an optimized plant growth trigger comprising of 1.5 mg/L *meta*-Topolin (*m*T), 5.0 mg/L of chitosan for multiple shoot induction along with 1.5 mg/L indole-3-butyric acid (IBA), and 5.0 mg/L phloroglucinol (PG) for rooting (Bhattacharyya et al., 2022). The cultures were maintained under optimum growth conditions at 25 ± 2°C temperature, 80% relative humidity, and 12 h alternative photoperiod of light with 40 μmol m^–2^ s^–1^ irradiance provided by cool white fluorescent light (Phillips, India). For transcriptome sequencing, leaf and pseudobulb tissues were excised from the fully developed *in vitro* grown plants.

### RNA extraction, library preparation, and sequencing

Total RNA was isolated following the protocol described by Li and Trick (2005). The quality and quantity of RNA were checked using a Qubit 2.0 Fluorometer (Invitrogen, United States) and NanoDrop 1000 (NanoDrop Technologies, United States). Libraries (paired-end) of leaf and pseudobulb tissues were prepared using TruSeq stranded mRNA sample prep kit v2 (Illumina Incorporation, United States). The libraries were quantified using Qubit 4.0 Fluorometer (Life Technologies, United States) and DNA 1000 chip on Bioanalyzer (Agilent 2100 technologies, United States). ExAMP chemistry was used to generate the clusters and 400 pM of each prepared library was loaded in an S2 flow cell lane of the 2 × 100 bp paired-end format of NovaSeq 6000 (Illumina Inc., United States). The raw sequenced data was de-multiplexed with a BCL2FASTQ converter which resulted in FASTQ data format of final sequence reads. The quality was checked using the Fast QC tool for final sequence reads and then subjected to filtering with the in-house developed tool filteR (Gahlan et al., 2012). Horizontal filtering was done to check the quality of each read, whereas vertical filtering was done to check the consistency of the quality score position in the read files. *De novo* assembly was performed for the reads which had a Phred score of ≥QV30 for more than 70% bases. The reads that did not fall under the “cutoff range” were removed.

### *De novo* sequence assembly

The *de novo* assembly of transcripts was done using the SOAP*de novo*-Trans with 127 k-mer value and default parameters; the optimal k-mer was found after running the assembly for various k-mer with an insert length of 32–260 bp. Based on parameters like average length of contigs, coverage, N50, and percentage length greater than 1,000 bp, the best assembly result was selected. TGICL-CAP3 clustering with 90% identity cut-off for each terminal joining of assembled transcripts and terminal length criteria of 40 bp for patching up of scaffolds was used. The resultant final contigs and singletons which did not fit TGICL-CAP3 criteria were then combined to serve as inputs for CD-HIT with a cut-off of 95% similarity. Based on clustering using CD-HIT-EST to eliminate redundancy and by using a set of in-house developed python scripts, the contigs that had no sequence similarity were subjected to DS clustering (Gahlan et al., 2012), and finally, the contigs that did not show any remarkable hits were further transformed into six open reading frames (ORFs). Reverse PSI-BLAST (RPS Blast) tool was used to search conserved domain database (CDD) for remaining contigs.

### Functional annotation of the assembled transcriptome

The annotation of finally assembled transcripts was done by similarity search against NCBI non-redundant (NR) database using the BLASTX algorithm. Databases like NCBI-nr, Uniprot, Gene Ontology (GO), Enzyme Classification (EC), and Kyoto Encyclopedia of Genes and Genomes (KEGG) were used for homology search. Multiple hits were obtained for maximum unigenes using the Annot8r tool. The top hits were chosen for each unigene based on the *E*-value cut-off of 10^–05^ and the best bit score obtained.

### Differential expression analysis

The reads obtained from each of the samples were mapped using Bowtie to count the number of mapped reads having default scope for 2 mismatches. The counts of distinctly mapped reads were obtained using an in-house developed PERL script. For differential expression analysis between two individual plant tissues, i.e., leaf and pseudobulb, EdgeR was employed. The input files for each sample consisted of a matrix of mapped reads which was followed by a one-to-one analysis using a *p*-value at ≤0.05 cut-off for the reads from each sample. Finally contigs with log FC ≥ +1 and *p*-value at <0.05 were considered as differentially expressed unigenes. Expression values in terms of fragments per kilobase per million (FPKM), GO, EC, and KEGG annotations were determined by transforming the count matrix files into FPKM by using in-house developed scripts. Heatmaps and figures were prepared using ggplots, cluster package, and Biobase in R for all the differentially expressed genes (DEGs). Using AgriGOv2.0^1^ (Tian et al., 2017), GO enrichment analyses were performed wherein GO enriched categories were identified using the Bonferroni multi-test adjustment method and the hygrogeometric statistical test at the significance level of 0.05. Transcripts encoding for transcription factors (TFs) in leaf and pseudobulb tissues were identified using the plant TFDBv5.0 database^2^ (Tian et al., 2020) and the genes involved in different primary and secondary metabolic processes were also identified using Plant Metabolic Network (PMN) database.^3^

### Estimation of β-sitosterol and eugenol content in leaf and pseudobulb

β-sitosterol and eugenol contents were estimated using hydrophilic interaction chromatography using an ACQUITY ultra-performance liquid chromatography (UPLC) BEH Shield RP18 (2.1 mm × 100 mm, 1.7 μm) column installed on UPLC system (Waters, India). Two-solvent systems (A; 0.05% formic acid in water and B; 0.1% formic acid in methanol) were used for the separation of samples with gradient programs; 10–20% B at 0–2 min, 20–40% at 2–4 min, 40–20% at 4–7 min, and 20–10% at 7–10 min. The column was equilibrated with the same mobile phase for 1 min at a flow rate of 0.25 μl min^–1^. The injection volume was 2 μl. Elution was monitored at 254 nm using a photo diode array (PDA) detector with column temperature maintained at 30°C. β-sitosterol and eugenol were identified by comparing their respective retention times and quantified using calibration curves prepared with standard compounds.

### Validation of RNA-seq data through reverse transcription quantitative polymerase chain reaction (RT-*q*PCR)

The robustness of transcriptome data was validated by using RT-*q*PCR. The extracted total RNA was reverse transcribed using a cDNA synthesis kit (Thermofisher Scientific, United States), following manufacturer’s instructions. Primers for the validation of the selected genes were synthesized using Primer Express 3.0.1 primer design tool (Invitrogen, United States) (Supplementary Table 1). RT-*q*PCR was performed with the following conditions: 10 min at 94°C (initial denaturation), 40 cycles of 94°C for 15 s (denaturation), 58–60°C (primer annealing) for 30 s followed by melt curve analysis at 60°C for 30 s (Quantstudio, Applied Biosystems, United States). *Actin* was used as an internal control gene. The expression of the genes was estimated using the 2^–ΔΔ^ ^CT^ method (Livak and Schmittgen, 2001), and the values were transformed (log_2_) to generate expression profiles.

**FIGURE 1:**
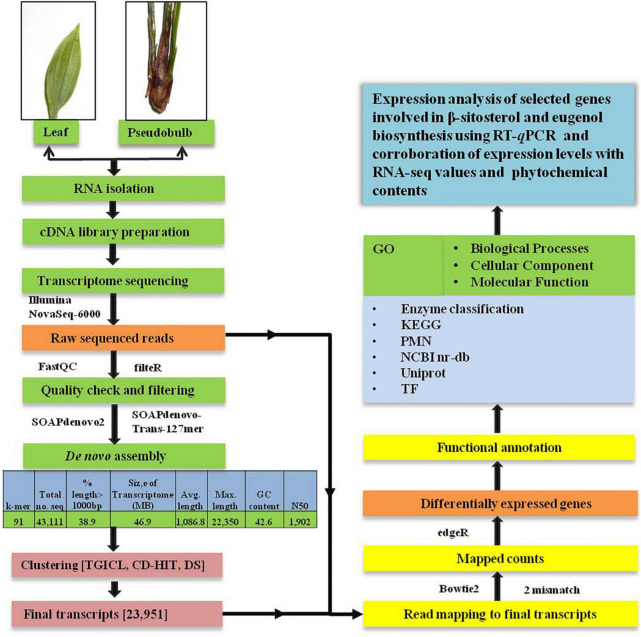
Schematic representation of workflow used for *de novo* transcriptome analysis and annotation of high throughput data of *M. acuminata*.

A schematic diagram representing the workflow for *de novo* analysis of the transcriptome data obtained from leaf and pseudobulb tissues of *M. acuminata* has been provided in Figure 1.

## Results and discussions

### Plant tissue culture of *M. acuminata*

**FIGURE 2:**
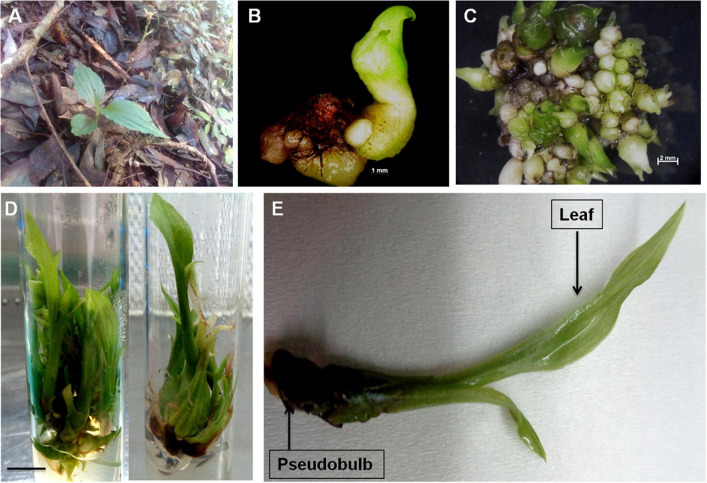
**(A)** Wild plants of *M. acuminata* growing in natural habitat; **(B)** initiation of aseptic cultures using transverse thin cell layer (*t*-TCL) explants (bar = 1 mm); **(C)** proliferation of protocorm like bodies (PLBs) from *t*-TCL explants and initiation of shoot buds (bar = 2 mm); **(D)** multiplication and growth of plants after 8 weeks of culture (bar = 1 cm); and **(E)** full grown plant of *M. acuminata* with differentiated organs.

Orchids are primarily valued for their attractive flowers (Bhattacharyya et al., 2019) and also for their use in various herbal systems of medicines (Bhattacharyya et al., 2019; Gantait et al., 2021). Commonly known as “*Jeevak*”, the pseudobulb of *M. acuminata* is used in the Indian traditional system of medicine. However, unchecked harvesting of plants from the wild imposes the risk of destruction of patched and scattered natural populations of *M. acuminata*. Keeping this into consideration, a sustainable PTC based protocol was developed to reduce pressure on the fragmented natural plant population. Combined usage of *m*T and chitosan at concentrations of 1.5 and 5.0 mg/L, respectively, induced healthy shoots of *M. acuminata*, which were further transferred to an optimized rooting medium supplemented with IBA and PG at 1.5 and 5.0 mg/L concentrations, respectively (Bhattacharyya et al., 2022; Figures 2A–E). The modulatory effect of the plant growth regulators especially *m*T and thidiazuron (TDZ) has been well documented for medicinal orchids *D. nobile*, *D. aphyllum*, *Habenaria edgeworthii*, and *Ansellia africana* (Giri et al., 2012; Bhattacharyya et al., 2016, 2018, 2020). In addition, synergistic application of plant biostimulants and plant growth regulators (PGRs) has been reported to effectively magnify the production of metabolites by stimulating the biosynthetic pathways (Conrath et al., 1989; Baskaran et al., 2016). In the present report, the combined impact of *m*T and chitosan has been studied for the first time. The study also elucidates how various tissues of an orchid can be effectively utilized under controlled *in vitro* conditions for the synthesis of desired biomolecules. Since no reports exist on the elucidation of biosynthetic pathways involved in secondary metabolite biosynthesis in *M. acuminata* and their subsequent corroboration with metabolite profiles, the present work endeavors to understand the involvement of the vital genes in metabolite biosynthesis through tissue-specific transcriptome analysis. The approach also opens an avenue for genetic manipulation *via* metabolic engineering.

### Transcriptome sequencing and *de novo* assembly

Tissue-specific transcriptome analysis is an established approach to gain valuable insights into the molecular mechanisms of secondary metabolite biosynthesis (Gantait et al., 2021). However, limited transcriptome data is available for orchids except for dendrobes (Dhiman et al., 2019). In *D. huoshanense*, the genes associated with the secondary metabolite biosynthesis in the leaf, stem and roots were identified by transcriptome analysis (Zhou et al., 2020). Transcriptome profiling revealed genes involved in isoflavonoid biosynthesis in different tissues of *Pueraria lobata*, an orchid species of medicinal importance (Wang et al., 2021). Being a medicinal orchid of biopharmaceutical importance, tissue-specific transcriptome profiling of *M. acuminata* was crucial to identify key genes and regulatory mechanisms governing biosynthesis and differential partitioning of secondary metabolites in *M. acuminata.* Since pseudobulb is widely used in herbal preparations and a previous report on metabolite profiling had revealed promising content of metabolites in leaf tissue of *M. acuminata* (Bose et al., 2017a), cDNA libraries prepared from leaf and pseudobulb tissues of *M. acuminata* plants were sequenced by using NovaSeq 6000 sequencing platform.

For knowing the number of reads, the read types (paired-end), GC content for individual libraries, possible contamination, and other issues, fast QC was used. The final *de novo* assembly of transcripts was obtained using the SOAP *de novo*-*Trans-*127 mer with default parameters. A total of 43,111 sequences covering 46.9 MB, an average length of 1,086.8 bp, a maximum length of 22,350 bp, GC content of 42.6% and an *N*50 value of 1.9 kb were obtained. These transcripts were clustered into contigs and scaffolds using TGICL and CDHIT followed by DS clustering for the contigs that had no sequence similarity but which belonged to a large gene’s part, resulting in 23,951 unigenes (Supplementary Datasheet 1). Mapping of the unigenes onto the KEGG pathways facilitated the identification of pathway-specific unigenes involved in the β-sitosterol and eugenol biosynthesis.

### Functional annotation of unigenes

**FIGURE 3:**
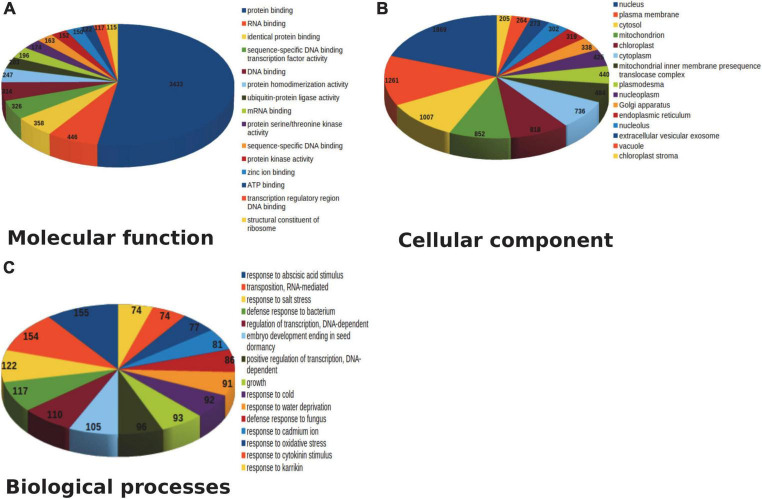
Top 15 pathway annotation of transcript in gene ontology (GO) database in *M. acuminata* **(A)** Molecular function; **(B)** Cellular component; **(C)** Biological process.

Amongst the generated transcriptome dataset, annotation of the assembled sequence reads derived from leaf and pseudobulb tissues of *M. acuminata* revealed that the mapped unigenes were categorized into GO categories. Out of 23,951 transcripts, 69.31% were annotated to cellular component categories, 68.46% to biological process, and 66.63% to molecular function categories. Enzyme classifications were found for 40.20% of transcripts, out of which 38.75% were assigned to biosynthetic pathways. The unannotated transcripts obtained through transcriptome data suggested a lack of information aligning with *M. acuminata*. Similarly, GO analysis underlined the functional heterogeneity amongst the documented transcriptome dataset where the maximum abundance of functional gene transcripts was grouped under cellular component and biological processes category followed by molecular function. The top 20 classes from these three categories are represented in Figure 3. In the molecular function category, genes for protein binding, RNA binding and sequence-specific DNA binding transcription factor activity were highly represented. In the biological process category, response to abscisic acid stimulus, defense response to the bacterium, and regulation of transcription were highly represented. Amongst cellular components, nucleus, plasma membrane, and cytosol were among the predominant categories.

### UPLC based estimation of β-sitosterol and eugenol content in leaf and pseudobulb

The medicinal properties of plants are attributed to the occurrence of various types of bioactive compounds of which steroids occupy an important position (Paul et al., 2017; Hu et al., 2022). The steroids are sub-classified into various groups of which phytosterols have attracted the attention of researchers due to their broad-spectrum medicinal properties (Hu et al., 2022). Structurally analogous to cholesterol, phytosterols are of universal occurrence within various members of the plant kingdom including orchids (Singh et al., 2014; Bin Sayeed et al., 2016; Aboobucker and Suza, 2019). Ethnomedicinally, *M. acuminata* is reported to possess bioactive molecules like β-sitosterol and eugenol (Bose et al., 2017a; Suyal et al., 2020). Amongst the two mentioned metabolites, β-sitosterol is a phytosterol that is well known for its therapeutic attributes including anti-cancerous, estrogenic, anti-diabetic, and anti-aging activities whereas eugenol is a phenylpropanoid, which is known to possess anti-inflammatory activity. Realizing the importance of these plant-derived metabolites in the pharmaceutical, food, and cosmetic industries, these can be produced *in vitro* systems through PTC based approach.

**FIGURE 4:**
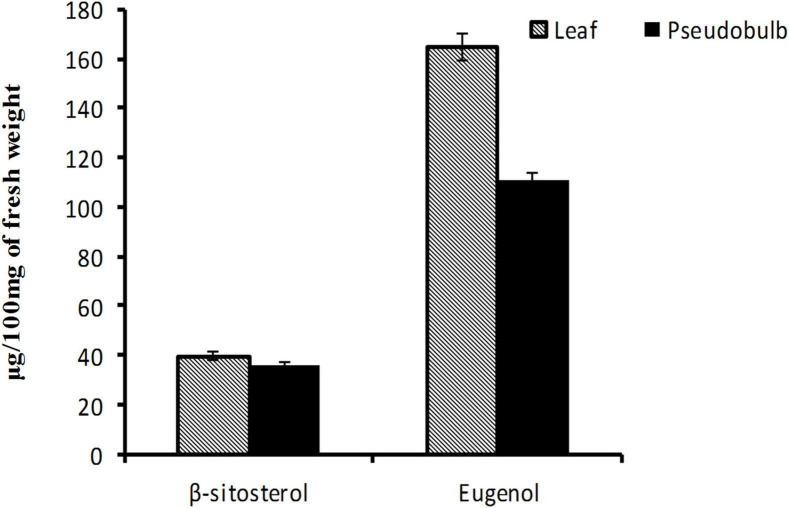
Eugenol and β-sitosterol content in leaf and pseudobulb of *in vitro* raised *M. acuminata.* Value in the bar represents mean of three independent biological replicates with standard error of mean.

In the present study, β-sitosterol and eugenol contents were determined in leaf and pseudobulb tissues of micropropagated *M. acuminata* by using UPLC (Supplementary Figure 1). Leaf tissue had promising content of both β-sitosterol (39.69 μg/100 mg) and eugenol (165.94 μg/100 mg) as compared to pseudobulb (β-sitosterol-36.25 μg/100 mg; eugenol-110.92 μg/100 mg), which is traditionally being utilized in herbal preparations (Figure 4). The present report is strongly corroborated with the findings of Bose et al. (2017a) in *M. acuminata*. Higher levels of β-sitosterol and eugenol in leaves suggested that leaves rather than pseudobulb may be used for medicinal purpose whereas, pseudobulb can be used for regeneration, thereby helping *in vitro* multiplication and conservation of *M. acuminata*.

### Identification and expression analysis of genes involved in β-sitosterol and eugenol biosynthesis

β-sitosterol is a bioactive phytosterol derived from isopentenyl pyrophosphate (IPP) and its isomer dimethylallyl diphosphate (DMAPP) formed *via* plastidic methylerythritol (MEP) and cytosolic mevalonate (MVA) pathways respectively (De-Eknamkul and Potduang, 2003; Suo et al., 2019). The expression pattern of genes involved in the MEP and MVA pathway was analyzed in both leaf and pseudobulb tissues (Supplementary Datasheet 2). Several enzymes of the MEP pathway, *1-deoxy-D-xylulose-5-phosphate synthase* (*dxs*), *dimethylallyltranstransferase* (*dmatt*), *4-*[*cytidine5*(*prime*)-diphospho]*-2-C-methyl-D-erythritol kinase* (*ispE*), *2-C-methyl-D-erythritol 2,4-cyclodiphosphate synthase* (*ispF*) were downregulated in pseudobulb as compared to that in leaf. *Diphosphomevalonate decarboxylase* (*mvdd*), *3-hydroxy-3-methylglutaryl coenzyme A synthase* (*hmgs*), and *hydroxymethylglutaryl-coenzyme A reductase* (*hmgr*) of the MVA pathway were also downregulated in pseudobulb as compared to that in leaf tissue. *Farnesyl diphosphate synthase* (*fpps*) and *cycloartenol synthase* (*cas*) were downregulated in pseudobulb as compared to that in leaf. Overall downregulation of enzymes involved in β-sitosterol biosynthesis corroborated with lower β-sitosterol content in pseudobulb as compared to that in leaf tissue.

Like phytosterols, eugenol and isoeugenol are bioactive phenylpropanoids synthesized by plants as attractants of pollinators or as defense compounds (Koeduka et al., 2006). Eugenol biosynthesis starts with amino acid phenylalanine which undergoes sequential nine enzyme-catalyzed steps to form eugenol (Singh et al., 2020). The expression of transcripts involved in eugenol biosynthesis was analyzed in leaf and pseudobulb. Amongst all the genes involved in eugenol biosynthesis, the expression of *phenylalanine ammonia-lyase* (*pal*), *cinnamyl alcohol dehydrogenase* (*cad*), and *cinnamyl alcohol reductase* (*ccr*) was similar in both the tissues. Similar expression of these genes was anticipated in both the tissues, as the enzymes coded by these genes play an important role in diverting metabolites toward the biosynthesis of phenylpropanoids and related compounds. However, transcripts encoding for *coumarate CoA ligase* (*4-cl*), *diacylglycerol O-acyltransferase* (*dgat*), and *alcohol acetyltransferase/2-acylglycerolO-acyltransferase* (*acetyl-tag*) were downregulated in pseudobulb as compared to that in leaf. *4-cl* and *dgat* are involved in the specific partitioning of metabolites towards eugenol biosynthesis. Downregulation of these enzymes in pseudobulb as compared to that in leaf tissue corroborated with higher eugenol content in leaf and further suggested that leaf rather than pseudobulb may be used for medicinal preparations.

### Analysis of differentially expressed genes in leaf and pseudobulb

**FIGURE 5:**
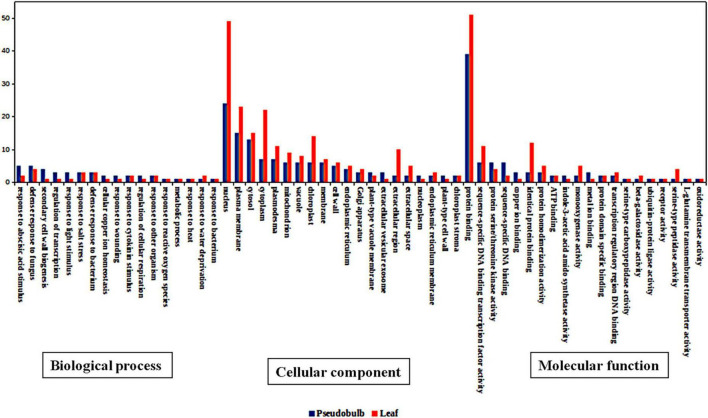
Top 20 gene ontology (GO) categories of differentially expressed genes in *M. acuminata* for biological process, cellular component, and molecular function.

The differentially expressed genes in leaf and pseudobulb were analyzed. In the biological process category, transcripts associated with response to wounding, defense response to fungus, cell growth, and response to abscisic and jasmonic acid stimulus were upregulated in pseudobulb as compared to that in leaf (Figure 5). Pseudobulb is responsible for the regeneration of *in vitro* and naturally occurring populations of *M. acuminata*. An upregulation of defense and plant hormone-related pathways was envisaged as these might play an important role in coping with various environmental stresses. Amongst cellular components, transcripts associated with the nucleus, cell membrane, and chloroplast were downregulated in pseudobulb as compared to that in leaf. In the molecular function category, protein binding and sequence-specific transcription factor binding activity were downregulated in pseudobulb as compared to that in leaf suggesting a high degree of protein interaction and transcription factor-mediated gene regulation in leaf as compared to that in pseudobulb (Figure 5).

**FIGURE 6:**
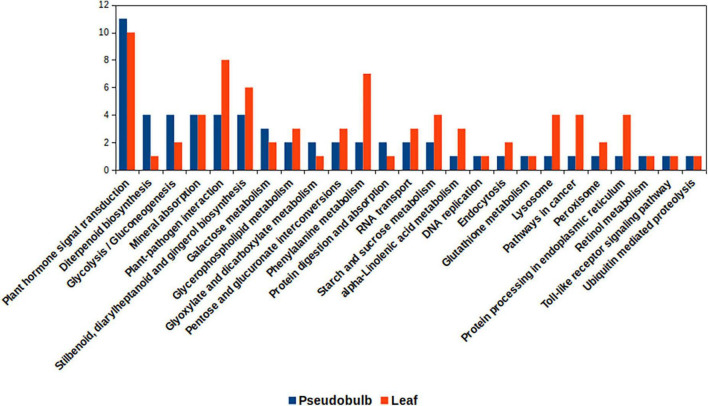
Kyoto encyclopedia of genes and genomes (KEGG) pathways associated with differentially expressed genes for leaf and pseudobulb in *M. acuminata.*

In the KEGG pathway category, transcripts related to starch and sucrose metabolism, phenylalanine metabolism, stilbenoid, diarylheptanoid, and gingerol biosynthesis were upregulated in leaf as compared to that in pseudobulb (Figure 6). Heatmaps depicted expression pattern of unigenes associated with differentially expressed KEGG pathways (Supplementary Figure 2 and Supplementary Datasheet 3). Phenylalanine being a substrate for biosynthesis of phenylpropanoids and related compounds, an upregulation of transcripts associated with phenylalanine metabolism in leaf corroborated with higher eugenol content in leaves. However, transcripts related to plant hormone signal transduction and diterpenoid biosynthesis were upregulated in pseudobulb as compared to that in the leaf. Upregulation of hormone signal transduction pathways in pseudobulb over leaf suggested an important role of phytohormones in mediating *in vitro* regeneration of *M. acuminata* plantlets from pseudobulb explants. The role of plant in hormones *in vitro* regeneration of plants is well documented. Also, chemicals like chitosan and *m*T have been reported to modulate phytohormone levels in plants (Bhattacharyya et al., 2021). Overall differential expression across the various KEGG pathways in leaf and pseudobulb suggested tissue-specific modulation of metabolic processes in *M. acuminata.*

### Transcription factors

**FIGURE 7:**
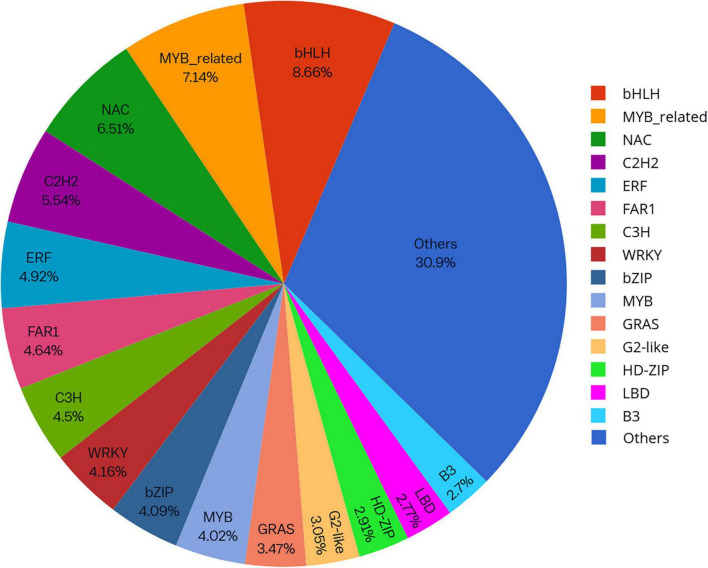
Distribution of top transcription factors (TFs) families identified in *M. acuminata.*

**FIGURE 8:**
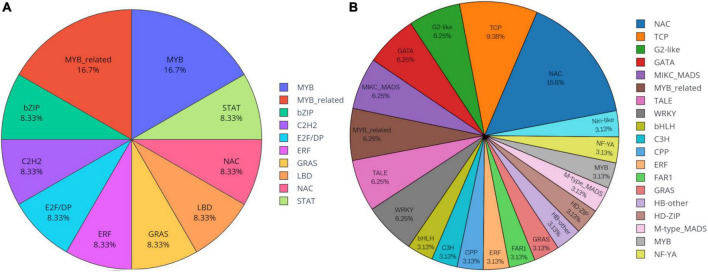
Differentially expressed transcription factors (TFs) in **(A)** pseudobulb and **(B)** leaf tissues of *M. acuminata*.

Knowledge of transcriptional regulation of secondary metabollite biosynthesis, along with an explanation of the biosynthetic pathways is much anticipated (Chezem and Clay, 2016; Singh et al., 2017). TFs regulate the expression of target genes by binding to specific *cis*-regulatory elements located in the promoter region of genes eventually regulating a vast network of biological and metabolic processes (Patra et al., 2013). In the present study, a total of 1,443 unigenes belonging to 56 TF families were identified as TFs (Figure 7). Analysis of differentially expressed gene datasets in leaf and pseudobulb of *M. acuminata* revealed that as compared to pseudobulb, higher numbers of TFs were expressed in leaf suggesting a high level of TF mediated gene regulation in the leaf. However, MYB, bHLH, GRAS, NAC, and ERF families were well represented in both leaf and pseudobulb tissue of *M. acuminata* (Figure 8 and Supplementary Datasheet 4). Amongst the various classes of TFs, MYB is one of the largest transcriptional regulators playing a key role in the regulation of secondary metabolism in medicinal plant species (Cao et al., 2020). Apart from MYB, the role of GRAS, bHLH, ERF, and NAC TFs in the regulation of biosynthesis of secondary metabolites is also well documented (Rohrmann et al., 2011; Zhao et al., 2013; Chezem and Clay, 2016; Duraisamy et al., 2016; Fu et al., 2017). Enrichment of these transcripts in both the tissues suggests their involvement in the biosynthesis of valuable metabolites (Figure 8).

Further, profiling of target metabolites (β-sitosterol and eugenol) by UPLC suggested that apart from predominance in the pseudobulb, metabolites were also present and accumulated in leaf tissues which are supported by the findings of Bose et al. (2017a) in *M. acuminata*. The present finding suggested that from pseudobulb, metabolites are possibly transported to the other tissues such as the leaf. Furthermore, the metabolite production can be further increased by the administration of a suitable elicitor in cell suspension cultures derived from leaf/pseudobulb protocorm like bodies (PLBs) or callus. This will pave the path for both conservations and commercial utilization. Similar findings were reported for *Achyranthes bidentata* and *T. govanianum*, also (Li et al., 2016; Singh et al., 2017).

### Identification of simple sequence repeats

**FIGURE 9:**
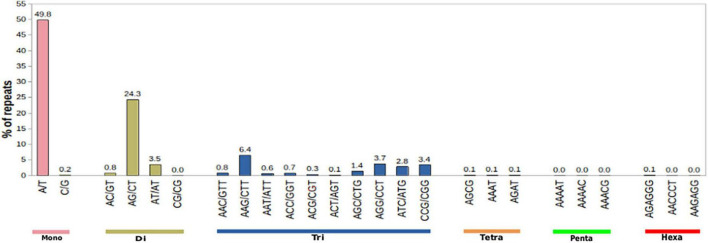
Distribution of simple sequence repeats (SSRs) in *M. acuminata* transcriptome.

Apart from providing critical insights into the pathways of secondary metabolite biosynthesis in medicinal plants, transcriptome sequencing also facilitates the detection of 2–6 base pair tandem repeat nucleotide motifs defined as SSR or microsatellites. EST-SSRs play a vital role in genetic breeding by providing a wealth of molecular markers and can be used for the construction of genetic maps in plants (Varshney et al., 2005a,b; Ellis and Burke, 2007; Kalia et al., 2011). These have been used as a resource for candidate markers in population genetic studies. SSRs were mined using the microsatellite identification tool (MISA) program with the following parameters: di- ≥ 6 nt, tri- ≥ 5 nt, tetra- ≥ 5 nt, penta- ≥ 4 nt, and hexa- ≥ 4 nt. A total of 5,370 SSRs were identified in 23,951 transcripts. Amongst the identified SSRs, the most abundant were mononucleotide repeat (11.20%) followed by di (6.43%) and tri (4.52%) nucleotide repeat types (Figure 9 and Supplementary Table 2). To date, no data exist on EST/SSRs and microsatellites of *M. acuminata* to the best of our knowledge. Being an endangered terrestrial orchid species of broad-spectrum therapeutic importance, knowledge of genetic diversity is pivotal in the formulation of effective conservation strategies for its sustainable utilization (Bhattacharyya and Van Staden, 2018). Therefore, the identification and development of highly polymorphic EST-SSRs can be used for distinguishing closely related populations of *M. acuminata* and could thereby aid in the identification of superior strains for improvement.

### Validation of transcriptome data by RT-*q*PCR

**FIGURE 10:**
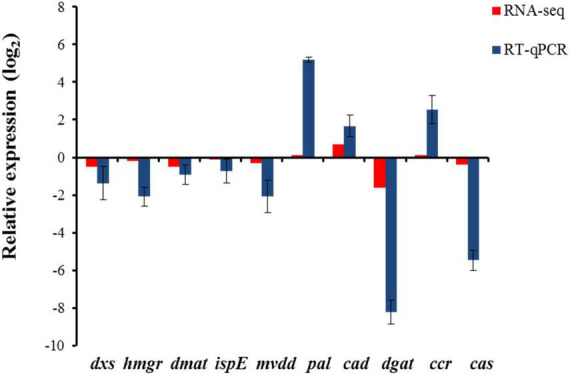
Expression of genes involved in biosynthesis of β-sitosterol and eugenol in *M. acuminata*. Relative expression ratios in pseudobulb have been calculated with reference to the leaf tissue. The relative expression values were log_2_ transformed. *dxs*, 1-deoxy-D-xylulose-5-phosphate synthase, *hmgr, hydroxy 3 methylglutaryl coenzyme A reductase*, *dmat*, *dimethylallyltranstransferase*, *ispE*, 4-diphosphocytidyl-2-C-methyl-D-erythritol kinase, *mvdd*, *diphosphomevalonate decarboxylase*, *pal*, *phenylalanine ammonia lyase*, *cad*, cinnamyl alcohol dehydrogenase, *dgat*, *diacylglycerol O-acyltransferase*, *ccr*, *cinnamoyl coA reductase*, *cas*, *cycloartenol synthase*. *Actin* was used as endogenous control to normalize the data. Each value in the bar diagram represents mean of three independent biological replicates and error bar represents standard error of mean.

Reverse transcription quantitative polymerase chain reaction was used to validate the expression of genes involved in β-sitosterol and eugenol biosynthesis as obtained from transcriptome data. The results revealed a significant correlation between the expression of genes obtained by RT-*q*PCR and RNA-seq data (Figure 10). Further, the expression patterns of the selected genes were correlated with the assayed metabolite fractions, i.e. β-sitosterol and eugenol. Higher expression of genes involved in β-sitosterol and eugenol biosynthesis and relatively higher content of target metabolites (β-sitosterol and eugenol) in leaf tissue as compared to pseudobulb was obtained. Data suggested that leaf tissues rather than pseudobulb can be used as an alternate source of bioactive metabolites in *M. acuminata*.

## Conclusion

Terrestrial orchids are largely unexplored medicinal plant species. The present report provides the first-ever insights into the tissue-specific transcriptome of *M. acuminata* (*Jeevak*), a key ingredient of *Ashtvarga* in *Ayurveda*. The *de novo* assembled transcriptome provides a comprehensive resource information for further research aimed at enhancing secondary metabolite biosynthesis in *M. acuminata*. The research findings also firmly establish the utility of *in vitro* propagules in providing an alternative source of metabolite prospection. The expression of genes involved in β-sitosterol and eugenol biosynthesis in leaf and pseudobulb corroborated with target metabolite contents and substantiates the utility of leaf tissues as a valuable alternative source for metabolites in *M. acuminata*. The present research approach can serve as a research model for the PTC-mediated conservation and RNA sequencing aided gene mining in non-model medicinal plants, especially orchids. Functional characterization of regulatory genes/enzymes/TFs involved in secondary metabolite biosynthetic pathways could facilitate a metabolic engineering program in *M. acuminata*.

## Data availability statement

The original contributions presented in this study are publicly available. This data can be found here: NCBI, PRJNA842241.

## Author contributions

PB contributed to PTC of *M. acuminata*, RNA extraction library preparation, and drafting of the manuscript. TS performed the RT-*q*PCR gene expression analysis, data analysis, and drafting of the manuscript. AY performed the bioinformatics analysis. LL assisted in RNA extraction and gene expression analysis. Ritu assisted in uploading the transcriptome data in SRA database also assisted in revising and rechecking of the bioinformatics data presented in the research manuscript. MS performed the transcriptome sequencing and assisted in library preparation. RJ performed UPLC profiling and helped in analyzing the phytochemical data. RS supervised bioinformatics analysis and finalized the manuscript. SK planning, conceptualization, overall supervision, and finalization of the manuscript.

## Footnotes

1
https://systemsbiology.cau.edu.cn/agriGOv2/c_SEA.php

2
http://planttfdb.cbi.pku.edu.cn/

3
https://plantcyc.org/
