# Supplementary material for: De novo transcriptome based insights into secondary metabolite biosynthesis in Malaxis acuminata (Jeevak)–A therapeutically important orchid
Source: Front Plant Sci. 2022 Oct 18;13:954467. doi: 10.3389/fpls.2022.954467 (PMC9623264; doi:10.3389/fpls.2022.954467)
Supplement: Supplementary file 1 [file Table_1.pdf]

Title: ***De novo*** **transcriptome based insights into secondary metabolite biosynthesis in** ***Malaxis acuminata*** **(*Jeevak*) – A therapeutically important orchid**

Paromik Bhattacharyya[1¥], Tanvi Sharma[1¥], Abhinandan Yadav[2¥], Lucy Lalthafamkimi [3, 5], Ritu [2, 4], Mohit Kumar Swarnkar[1], Robin Joshi[4], Ravi Shankar[2,5*] and Sanjay Kumar[1,5*]

[1]Biotechnology Division, Council of Scientific and Industrial Research-Institute of Himalayan Bioresource Technology, P.O. Box 6, Palampur (H.P.) 176061, India

[2]Studio of Computational Biology & Bioinformatics, The Himalayan Centre for High-throughput Computational Biology, (HiCHiCoB, A BIC supported by DBT, India), CSIR-Institute of Himalayan Bioresource Technology (CSIR-IHBT), Palampur (HP), 176061, India

[3]Agrotechnology and Rural Development Division (ARDD), CSIR-North East Institute of Science & Technology, Jorhat 785006, Assam, India

[4]Chemical Technology Division, CSIR-Institute of Himalayan Bioresource Technology, Palampur, Himachal Pradesh 176061, India

[5]Academy of Scientific and Innovative Research (AcSIR), Ghaziabad - 201002, Uttar Pradesh, India

E-mails: sanjaykumar@ihbt.res.in (S. Kumar*) [Corresponding author]; ravish9@gmail.com (R. Shankar*) [co-corresponding author]

¥ Shares equal credit

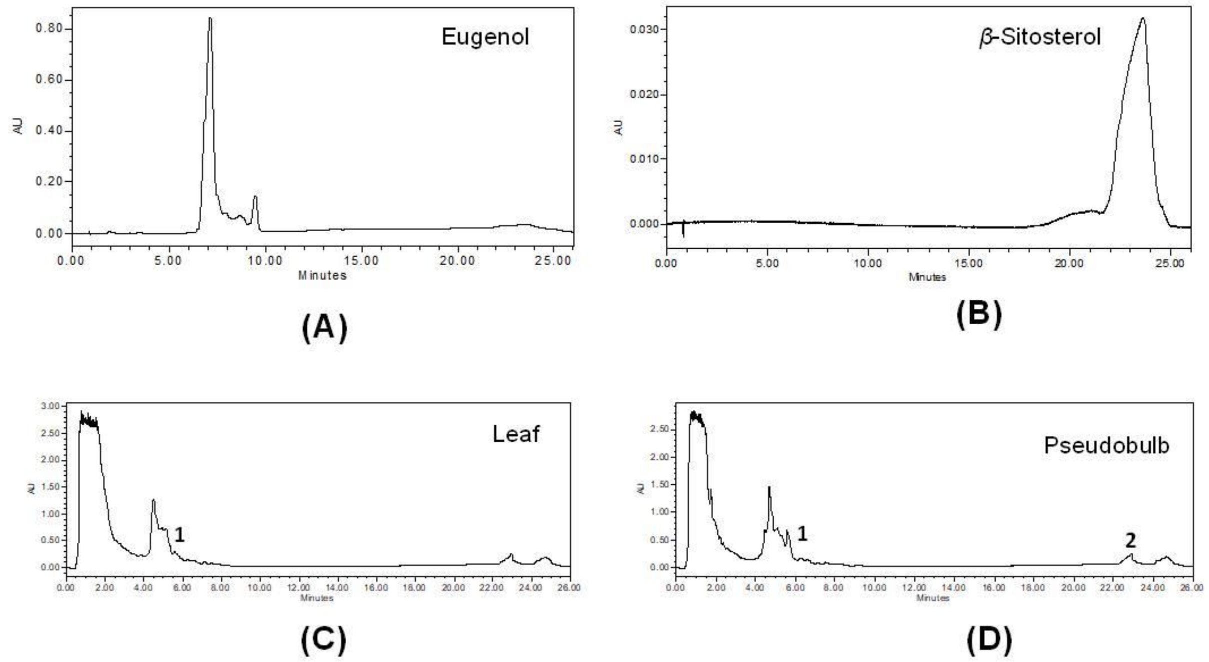

Fig S1 UPLC chromatograms of standards for (A) eugenol and (B) β-sitosterol ; Contents of β-sitosterol and eugenol content in leaf (C) and pseudobulb (D) of *M. acuminata*.

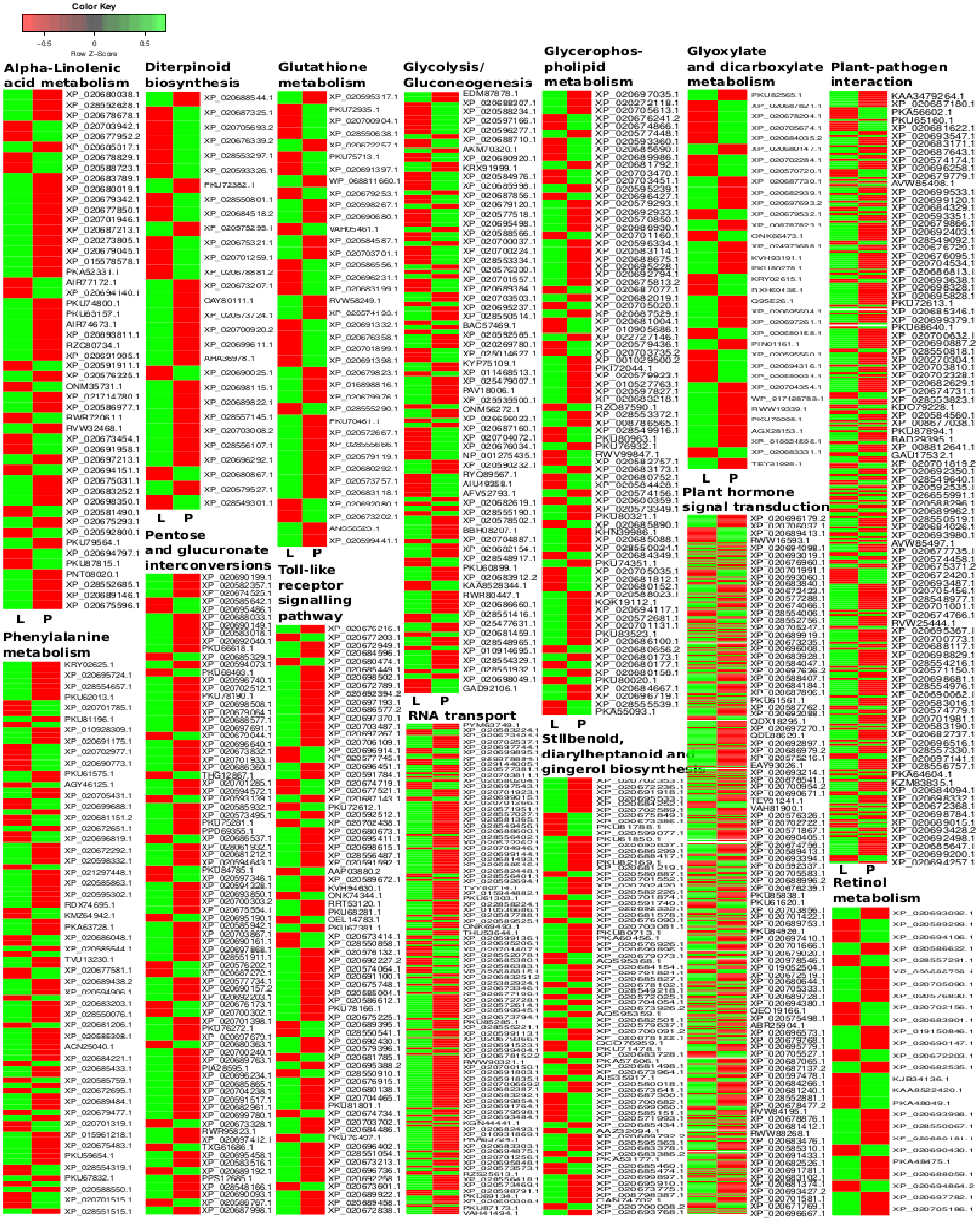
Color Key
-0.5
0
0.5
Row Z-Score
Alpha-Linolenic acid metabolism
Diterpinoid biosynthesis
Glutathione metabolism
Glycolysis/ Gluconeogenesis
Glycerophos-pholipid metabolism
Glyoxylate and dicarboxylate metabolism
Plant-pathogen interaction
Phenylalanine metabolism
Pentose and glucuronate interconversions
Toll-like receptor signalling pathway
RNA transport
Stilbenoid, diarylheptanoid and gingerol biosynthesis
Plant hormone signal transduction
Retinol metabolism
L
P

Fig S2 Heatmaps of selected unigenes associated with differentialy expressed KEGG pathways in *M. acuminata.* P and L representing pseudobulb and leaf respectively.

Supplementary Table S1 Primer sequences used for expression analysis of genes involved in β-sitosterol and eugenol biosynthesis in *M. acuminata*.

S No.	Gene name	Contig ID	Primer sequence	Tm (°C)
1.	1-deoxy-D-xylulose-5-phosphate synthase (*dxs*)	C114706	Fwd:CCAGGTGGTGCACGATGTC<br>Rev:GCCATGTATGCGATGTCGAA	59
2.	Dimethylallyltranstransferase (*dmat*)	scaffold4048	Fwd: CCGAGAATCTTCGGTGAAGCT<br>Rev: AAGCGAACGGGAAGATGATG	59
3.	4-(cytidine5{prime}-diphospho)-2-C-methyl-D-erythritolkinase (*ispE*)	CL1362 Contig1	Fwd:GCAGCTTCAATATCACGAACAGA<br>Rev: TGACGCGCAAGAAATGATTC	58
4.	Diphosphomevalonate decarboxylase (*mvdd*)	C112922	Fwd: CCTCGCCTCTCATCCTTGAA<br>Rev: GGCTCAATGGGAAGGAGATTC	59
5.	Hydroxymethylglutaryl-CoA reductase (*hmgr*)	scaffold2044	Fwd: GGAGGACAGTAGCTCCCGTTT<br>Rev: TGCCTCATGGCTCGATGAC	58
6.	Cycloartenol synthase (*cas*)	scaffold10597	Fwd: AGACGCTGGACAGGCTGAAA<br>Rev: GGAAATTCACCACTCTCCAGTTG	59
7.	Phenylalanine ammonia-lyase (*pal*)	C112964	Fwd: CGGAGTAGCCTTGGAGGAGAGT<br>Rev: GCAGGGATCTTCGGAATCAA	60
8.	Cinnamyl alcohol dehydrogenase (*cad*)	C100210	Fwd: AAGTACGCCGGAGTCGTCTCT<br>Rev: ATTAATGGCGGTGACGCAAA	59
9.	Cinnamoyl-CoA reductase (*ccr*)	C100900	Fwd: GCACACAGCCTAGCCAGTGA<br>Rev: ATGCCTTGGTCCCCTGTTG	59
10.	Diacylglycerol -O-acyltransferase (*dgat*)	C101438	Fwd: TCTACGGTTGCCCTCTTTCTCT<br>Rev: CAATAACTGCAGCGCCAAGAC	58

Supplementary Table S2. Simple sequence repeats (SSRs) in *M. acuminata* transcriptome

Definement of microsatellites (unit size / minimum number of repeats):	(1/10) (2/6) (3/5) (4/5) (5/5) (6/5)
Maximal number of bases interrupting 2 SSR microsatellite	100
**Results of microsatellite search**	
Total number of sequences examined	23951
Total size of examined sequences (bp):	32819618
Total number of identified SSRs	5370
Number of SSR containing sequences	4356
Number of sequences containing more than 1 SSR:	813
Number of SSRs present in compound formation:	296
**Distribution to different repeat type classes**	
**Unit size**	**Number of SSRs**
1.	2682
2.	1539
3.	1082
4.	27
5.	11
6.	29
